# Empagliflozin Inhibits IL-1β-Mediated Inflammatory Response in Human Proximal Tubular Cells

**DOI:** 10.3390/ijms22105089

**Published:** 2021-05-11

**Authors:** Markus Pirklbauer, Sebastian Sallaberger, Petra Staudinger, Ulrike Corazza, Johannes Leierer, Gert Mayer, Herbert Schramek

**Affiliations:** Department of Internal Medicine IV–Nephrology and Hypertension, Medical University Innsbruck, Anichstrasse 35, 6020 Innsbruck, Austria; sebastian.sallaberger@hotmail.com (S.S.); petra.staudinger@tirol-kliniken.at (P.S.); ulrike.corazza@tirol-kliniken.at (U.C.); johannes.leierer@i-med.ac.at (J.L.); gert.mayer@i-med.ac.at (G.M.); herbert.schramek@aon.at (H.S.)

**Keywords:** SGLT2 inhibition, empagliflozin, renal inflammation, human proximal tubulus, pathway annotation analysis

## Abstract

SGLT2 inhibitor-related nephroprotection is—at least partially—mediated by anti-inflammatory drug effects, as previously demonstrated in diabetic animal and human studies, as well as hyperglycemic cell culture models. We recently presented first evidence for anti-inflammatory potential of empagliflozin (Empa) under normoglycemic conditions in human proximal tubular cells (HPTC) by demonstrating Empa-mediated inhibition of IL-1β-induced MCP-1/CCL2 and ET-1 expression on the mRNA and protein level. We now add corroborating evidence on a genome-wide level by demonstrating that Empa attenuates the expression of several inflammatory response genes in IL-1β-induced (10 ng/mL) normoglycemic HPTCs. Using microarray-hybridization analysis, 19 inflammatory response genes out of >30.000 human genes presented a consistent expression pattern, that is, inhibition of IL-1β (10 ng/mL)-stimulated gene expression by Empa (500 nM), in both HK-2 and RPTEC/TERT1 cells. Pathway enrichment analysis demonstrated statistically significant clustering of annotated pathways (enrichment score 3.64). Our transcriptomic approach reveals novel genes such as *CXCL8/IL8, LOX, NOV, PTX3*, and *SGK1* that might be causally involved in glycemia-independent nephroprotection by SGLT2i.

## 1. Introduction

SGLT2 inhibitors (SGLT2i) convey nephroprotection in type two diabetic patients with and without chronic kidney disease (CKD) [1,2,3,4]. Renal benefit has been primarily attributed to metabolic and hemodynamic drug effects, such as lowering of bodyweight, plasma volume, systemic blood pressure, and glomerular filtration pressure, as well as reducing renal hypoxia, rather than SGLT2i-mediated HbA1c-lowering [5]. In this regard, large clinical trials recently demonstrated renal benefit of SGLT2i irrespective of the presence or absence of diabetes mellitus in CKD [6] and heart failure [7] patients. Underlying molecular mechanisms for glycemia-independent protective effects of SGLT2i are still incompletely understood. Renal tissue inflammation, being directly involved in the pathogenesis of both diabetic and non-diabetic CKD, might play an important role in this context [8]. Indeed, anti-inflammatory potential of SGLT2i has been previously demonstrated in diabetic animal [9,10,11] and human studies [12,13], as well as hyperglycemic cell culture models [14]. In diabetic animal models, these SGLT2i-mediated anti-inflammatory effects were related to a decrease in glomerular and tubulo-interstitial damage [10,15]. This, however, has not been demonstrated under normoglycemic conditions until recently. In our own lab, we found an inhibitory effect of empagliflozin (Empa) on IL-1β-induced MCP-1/CCL2 and endothelin-1 expression on the mRNA and protein level under normoglycemic conditions in two independent human proximal tubular cells (HPTC) lines, namely HK-2 and RPTEC/TERT1. Both genes play an important role in early (inflammatory) pathogenesis of diabetic and non-diabetic kidney disease [16,17,18,19,20,21]. Genetic and pharmacologic inhibition of these two genes already demonstrated to convey beneficial renal effects in animal models [16,18,22,23,24,25,26] and humans [25,27,28,29,30,31] with diabetic and non-diabetic kidney disease. Most recently, Maayah and coworkers presented corroborating evidence for anti-inflammatory potential of Empa irrespective of glycemic effects by demonstrating a survival benefit among Empa-treated mice with LPS-induced septic shock. This was at least partially attributed to a suppression of renal and systemic inflammation, as well as a reduced rate of acute kidney injury [32]. We now aimed at evaluating the inhibitory effect of Empa on IL-1β-stimulated gene expression on the transcriptome level. Here we present novel evidence for anti-inflammatory potential of SGLT2i by demonstrating that Empa attenuates the expression of several inflammatory response genes (vide infra) in IL-1β-induced (10 ng/mL) normoglycemic HPTCs, namely HK-2 and RPTEC/TERT1 cells. Our transcriptomic approach presents novel genes such as *CXCL8/IL8, LOX, NOV, PTX3*, and *SGK1*, that might be causally involved in glycemia-independent nephroprotection by SGLT2i.

## 2. Results

While the effect of Empa on basal gene expression has been recently described in normoglycemic HPTCs by our lab [33], we present novel genome-wide evidence for Empa-mediated inhibition of inflammatory response in IL-1β-stimulated normoglycemic HPTCs. Microarray hybridization analysis identified 19 inflammatory response genes out of >30.000 human genes that present a uniform expression pattern, that is, inhibition of IL-1β (10 ng/mL)-stimulated gene expression by Empa (500 nM), in both HK-2 and RPTEC/TERT1 (Table 1AB) cells (*n* = 2, each) (Table 1). Additionally, we report only marginal effects of Empa (500 nM) on *SGLT2* gene expression in IL-1β (10 ng/mL)-stimulated HK-2 and RPTEC/TERT1 cells (Table 2).

Based on these 19 genes-of-interest, pathway enrichment analysis (DAVID bioinformatics database) demonstrated statistically significant clustering of 30 annotated pathways (enrichment score 3.64) (Table 3).

## 3. Discussion

Inflammatory pathways play an important role during the development and progression of diabetic and non-diabetic CKD [8,34]. Circulating markers of systemic inflammation have been associated with renal damage [18]. Pro-inflammatory cytokines and reactive oxygen species are secreted by proliferating proximal tubular cells during initial CKD pathogenesis, thereby leading to inflammatory cell recruitment and activation within the glomerular and tubulo-interstitial compartment [35,36]. Local renal inflammation and thus kidney damage is further aggravated by the release of IL-1β from inflammatory cells upon inflammosome activation [37]. Interestingly, anti-IL-1β strategies already demonstrated beneficial renal effects in animal models of CKD [38,39]. Our own lab previously presented first evidence for anti-inflammatory effects of SGLT2i in normoglycemic HPTCs by demonstrating Empa-mediated inhibition of IL-1β-induced MCP-1/CCL2 and ET-1 expression at the mRNA and protein level [33]. Here we show novel evidence for anti-inflammatory potential of SGLT2i on the transcriptome level by demonstrating that Empa attenuates the expression of 19 inflammatory response genes in IL-1β-induced (10 ng/mL) normoglycemic HPTCs (Table 1). In line with recent findings in a murine animal model [32], pathway enrichment analysis of these genes-of-interest revealed that “cellular response to LPS” is among the Empa-regulated pathway cluster (Table 3). Based on the available evidence for involvement in kidney disease pathogenesis, five out of these 19 inflammatory response genes are discussed in more detail below:

### 3.1. Empa-Mediated Inhibition of IL-1β-Induced CXCL Isoform 1, 2, 5 and 8 (= IL8) Expression

Chemokines are chemotactic cytokines that have been demonstrated to play a fundamental role in orchestrating inflammation, and as such, are involved in experimental and human (inflammatory) kidney diseases. Chemokines are expressed by all types of renal cells, including HPTCs, upon pro-inflammatory cytokine stimulation (e.g., IL-1β or TNF-α). By regulating the process of leukocyte trafficking to the site of inflammation, chemokines control both acute and chronic inflammation that is crucial during development and progression of CKD and interstitial fibrosis [40,41]. For example, urinary and serum levels of CXCL8/IL8 are elevated in diabetic kidney disease [42], and CXCL1 has been described to be associated with interstitial fibrosis in progressive IgA nephropathy [43]. Chemokine antagonism has been shown to attenuate renal injury in various animal models [40,44,45,46]. Thus, it is tempting to speculate that Empa-mediated inhibition of tubular chemokine expression adds to the nephroprotective, anti-inflammatory potential of SGLT2i.

### 3.2. Empa-Mediated Inhibition of IL-1β-Induced LOX Expression

Lysyl oxidase (LOX) is a cellular amine oxidase facilitating cross-linking between collagen and elastin by catalyzing peptidyl lysine oxidation [47]. Excessive accumulation of these insoluble, cross-linked proteins leads to ECM fibrosis and remodeling. Both circulating and tissue levels of LOX are increased in patients with organ fibrosis, for example, in the heart, lung, and liver. Besides fibrotic diseases, altered LOX activity is related to inflammatory diseases, as well as cancer progression [48]. It has been previously speculated that *LOX* might be a potential therapeutic target in fibrotic diseases and cancer; however, specific treatment options are missing. Recently, elevated serum and tissue LOX levels have been described to strongly correlate with the presence and degree of kidney fibrosis in CKD patients. Serum LOX levels have been suggested to serve as non-invasive diagnostic biomarkers for renal fibrosis [49]. Further studies are mandatory to confirm this association and to investigate a potential causal relationship between *LOX* expression and kidney fibrosis. Based on these data and in line with our previous findings demonstrating a direct anti-fibrotic effect of SGLT2i in 2 independent HPTCs [50], it is tempting to speculate that Empa-mediated inhibition of IL-1β-mediated *LOX* expression might exert nephroprotective effects. 

### 3.3. Empa-Mediated Inhibition of IL-1β-Induced NOV Expression

The nephroblastoma overexpressed (*NOV/CCN3*) gene is a member of the CCN family of matricellular proteins that play an important role during inflammation, tissue repair and cancer [51]. Genetic knockdown studies recently demonstrated that a reduced *NOV* expression in a murine model of progressive obstructive nephropathy limits inflammation as well as renal interstitial fibrosis compared to wild-type mice [52]. This animal model is corroborated by biopsy findings that demonstrate an increased tubulo-interstitial *NOV* expression in patients with tubulo-interstitial nephritis [52]. While this is the first report of a beneficial effect of *NOV* downregulation with respect to inflammation and renal fibrosis, previous studies demonstrated contradictory results by showing anti-inflammatory [53] and anti-fibrotic [54] *NOV* effects. The anti-inflammatory potential of *NOV* inhibition, however, is further corroborated by the fact that systemic overexpression of *NOV* increases MCP-1/CCL2 expression among nephritic rats [55]. It has been speculated that *NOV* might exert both pro- and anti-fibrotic effects depending on different cellular adaptive processes in experimental models [52]. It remains to be elucidated whether Empa-mediated downregulation of *NOV* mechanistically adds to the anti-inflammatory and anti-fibrotic potential of SGLT2i.

### 3.4. Empa-Mediated Inhibition of IL-1β-Induced PTX3 Expression

Pentraxins are multimeric pattern recognition proteins that can be divided into short (e.g., CRP and serum amyloid A) and long (e.g., pentraxin-3) isoforms. Pentraxin-3 (*PTX3*) is expressed in response to pro-inflammatory signals (e.g., IL-1, TNF-a, TLR-activation, LPS) by a variety of tissue and cell types, including innate immunity, as well as endothelial cells [56]. PTX3 is a multifunctional acute phase protein that is elevated in cardiovascular, autoimmune, and pulmonary disease [57,58,59], and has been described as independent marker of inflammatory activity [60,61]. PTX3 production has already been demonstrated in HPTCs [62], and elevated PTX3 expression levels have been documented in mesangial cells of patients with IgA nephropathy [63]. It has been speculated that PTX3 augments the renal inflammatory response as well as the atherogenic process during CKD [64]. PTX3 correlates negatively with GFR and is associated with cardiovascular disease, inflammation, and protein-energy wasting [64]. In CKD patients, PTX3 levels independently predict all-cause mortality [64]. It remains to be elucidated whether PTX3 is only a marker of renal inflammation or causally involved in CKD pathogenesis. Further mechanistic studies are mandatory to evaluate if an Empa-mediated inhibition of IL-1β-stimulated tubular *PTX3* expression conveys nephroprotective effects or not. Interestingly, patients with IgA nephropathy (that have been demonstrated to present with high PTX3 levels) were highly represented among those non-diabetic CKD patients that showed a SGLT2i-mediated benefit [6].

### 3.5. Empa-Mediated Inhibition of IL-1β-Induced SGK1 Expression

Serum and glucocorticoid-induced kinase 1 (*SGK1*) is a ubiquitously expressed, cell-volume-regulated gene stimulated by various growth factors and hormones, including mineralo- and glucocorticoids. *SGK1* regulates transepithelial ion transport by orchestrating trafficking of ion channels and transporters, thereby increasing both sodium reabsorption and potassium excretion [65,66]. It has been implicated in blood pressure control as well as the development of inflammatory and fibrotic processes [67]. Genetic or pharmacologic inhibition of *SGK1* attenuates renal inflammation, proteinuria, and fibrosis in response to mineralocorticoids [68,69]. Its pleiotropic role in kidney disease is further supported by the fact that SGK1 is associated with aldosterone-induced oxidative stress and podocyte injury [70]. Being expressed in antigen-presenting T-cells, it has been implicated in renal inflammatory processes [71]. Increased SGK1 activity has been suggested to play a pathophysiologic role in kidney damage and CKD progression both in the presence and absence of hypertension [66]. It has been previously speculated that *SGK1* might be a potential target to slow CKD progression [66]. By demonstrating an EMPA-mediated inhibition of IL-1β-induced *SGK1* expression, this effect might be a potential explanation for the positive clinical effect seen with the use of SGLT2i in CKD patients irrespective of the presence and absence of diabetes.

In summary, our transcriptomic approach reveals 19 inflammatory response genes, such as *CXCL8/IL8, LOX, NOV, PTX3*, and *SGK1*, that might be causally involved in glycemia-independent nephroprotection by SGLT2i. Further molecular analyses on mRNA and protein level are mandatory to confirm our transcriptomic findings in HPTCs. Given the lack of data regarding SGLT2i effects at the human tissue level, it has been previously stated that kidney biopsy studies among SGLT2i-naive vs. SGLT2i-treated patients are inevitable to confirm a causal relationship between anti-inflammatory and nephroprotective potential of SGLT2i. In this regard, primary inflammatory kidney diseases, such as IgA nephropathy and acute interstitial nephritis, have been proposed to be most suitable for these future clinical trials [72]. 

## 4. Materials and Methods

### 4.1. Cell Culture

Human kidney 2 (HK-2) cells were purchased from American Type Culture Collection (Rockville, MD, USA) and RPTEC/TERT1 cells from Evercyte GmBH (Vienna, Austria). HPTCs were cultured under normoglycemic conditions as previously described in detail [73,74,75,76,77]. Cell culture media were obtained from Gibco Life Technologies (Paisley, UK) via Thermo Fisher Scientific (Waltham, MA, USA), and supplements and reagents from Sigma Aldrich Productions GmbH (Steinheim, Germany) and Roche (Basel, Switzerland). Empa was obtained from Selleckchem (Houston, TX, USA) and recombinant human IL‑1β from R&D Systems (Minneapolis, MN, USA) via Biomedica Medizinprodukte GmbH (Vienna, Austria). Empa was solubilized in dimethyl sulfoxide (DMSO) to a stock solution of 500 yM and diluted for further experiments to a final concentration of 500 nM (1:1000) containing <0.1% DMSO. IL-1β was solubilized in phosphate-buffered saline (PBS) to a stock solution of 10 yg/mL and diluted for further experiments to a final concentration of 10 ng/mL (1:1000). After 24 h serum and supplement starvation and an additional medium change, cells were either used for experiments or left untreated (control). Then, 24 h stimulation with IL‑1β (10 ng/mL) and the combination of IL-1β (10 ng/mL) and Empa (500 nM) was performed in the absence of any serum and growth supplements. As described previously [50], a MTT (3-(4,5-dimethylthiazol-2-yl)-2,5-diphenyltetrazolium bromide) assay (Cell Proliferation Kit I, Roche Diagnostics GmbH, Penzberg, Germany) was used to assess cell viability for each experimental condition according to manufacturer instructions. No significant difference in cell viability was observed between the experimental conditions.

### 4.2. Microarray Hybridization Analysis

Agilent oligonucleotide microarrays were used according to the Agilent One-Color Microarray protocol (Agilent Technologies, Santa Clara, CA, USA). A detailed description has been published previously [50]. Briefly, 200 ng of total RNA isolated from HPTCs by using the innuPREP RNA Mini Kit 2.0 (Analytic Jena AG, Jena, Germany) was used for hybridization. Microarray hybridization analysis was conducted in biological duplicates for each experimental condition and followed the Minimum Information About a Microarray Experiment (MIAME) guidelines [78]. The complete microarray data sets are available at Gene Expression Omnibus (GEO) (http://www.ncbi.nlm.nih.gov/geo/) (accession numbers: GSE155867 and GSE169683).

### 4.3. Pathway Enrichment Analysis

Pathway enrichment analysis was conducted for differentially expressed genes in HPTCs (i.e., IL-1β-induced genes inhibited by Empa) by using the Database for Annotation, Visualization and Integrated Discovery (DAVID) (https://david.ncifcrf.gov/). This bioinformatics database provides functional gene annotation by utilizing a biological knowledgebase and analytic data-mining tools to systematically extract biological meaning from large gene lists [79].

### 4.4. Statistical Analysis

The expression analysis systematic explorer (EASE) score, a statistical test for over-representation of annotation classes, was calculated to statistically evaluate each annotation term. Statistically significant over-representation of annotation classes is reflected by an EASE score ≤0.05. The overall enrichment score, that is, the geometric mean of all annotation term *p*-values within an annotation cluster, was calculated to reflect biological significance [79].

## Figures and Tables

**Table 1:** Inflammatory response genes attenuated by empagliflozin in two independent human proximal tubular cell lines, namely HK-2 (A) and RPTEC/TERT1 (B).

A	HK-2 (Experiment 1)			HK-2 (Experiment 2)		
Gene Name	Fold Change to Control		Fold Change to IL-1β	Fold Change to Control		Fold Change to IL-1β
	IL-1β	IL-1β + EMPA	IL-1β + EMPA	IL-1β	IL-1β + EMPA	IL-1β + EMPA
ATP binding cassette subfamily A member 12 (ABCA12)	2.23	1.83	−0.40	2.39	1.28	−1.11
Rho GTPase activating protein 22 (ARHGAP22)	2.17	1.57	−0.60	1.81	0.89	−0.93
CD82 molecule (CD82)	2.43	1.14	−1.29	3.35	2.86	−0.50
colony stimulating factor 2 (CSF2)	4.52	3.43	−1.09	4.13	3.53	−0.60
C-X-C motif chemokine ligand 1 (CXCL1)	6.89	6.81	−0.08	3.95	3.34	−0.61
C-X-C motif chemokine ligand 2 (CXCL2)	7.17	6.92	−0.25	4.65	4.13	−0.52
C-X-C motif chemokine ligand 5 (CXCL5)	5.30	5.29	−0.02	2.98	2.63	−0.35
C-X-C motif chemokine ligand 8 (CXCL8)	6.29	6.21	−0.08	4.09	3.71	−0.38
insulin induced gene 1 (INSIG1)	3.97	3.87	−0.09	4.52	3.88	−0.65
lysyl oxidase (LOX)	1.74	1.29	−0.45	1.80	0.65	−1.15
nephroblastoma overexpressed (NOV)	1.90	0.62	−1.27	2.37	0.75	−1.63
prolyl 4-hydroxylase subunit alpha 2 (P4HA2)	2.56	2.12	−0.44	2.27	1.41	−0.86
Pim-2 proto-oncogene, serine/threonine kinase (PIM2)	2.97	2.92	−0.05	3.09	2.89	−0.19
pentraxin 3 (PTX3)	3.54	2.43	−1.11	2.87	2.17	−0.70
serum/glucocorticoid regulated kinase 1 (SGK1)	2.14	1.46	−0.67	1.91	0.76	−1.15
solute carrier organic anion transporter family member 2A1 (SLCO2A1)	2.04	1.78	−0.26	1.62	−0.04	−1.66
solute carrier organic anion transporter family member 4A1 (SLCO4A1)	5.59	4.86	−0.73	1.74	1.07	−0.67
superoxide dismutase 2 (SOD2)	5.92	5.83	−0.09	2.41	2.21	−0.20
tissue factor pathway inhibitor 2 (TFPI2)	3.70	3.22	−0.47	2.25	1.69	−0.57
**B**	**RPTEC/TERT1 (Experiment 1)**			**RPTEC/TERT1 (Experiment 2)**		
**Gene Name**	**Fold Change to Control**		**Fold Change to IL-1β**	**Fold Change to Control**		**Fold Change to IL-1β**
	**IL-1β**	**IL-1β + EMPA**	**IL-1β + EMPA**	**IL-1β**	**IL-1β + EMPA**	**IL-1β + EMPA**
ATP binding cassette subfamily A member 12 (ABCA12)	2.11	1.29	−0.82	3.90	3.23	−0.67
Rho GTPase activating protein 22 (ARHGAP22)	1.93	0.79	−1.14	2.94	2.27	−0.68
CD82 molecule (CD82)	3.70	2.99	−0.71	2.65	1.42	−1.24
colony stimulating factor 2 (CSF2)	3.98	3.50	−0.49	6.95	5.85	−1.11
C-X-C motif chemokine ligand 1 (CXCL1)	3.71	2.96	−0.76	7.62	7.53	−0.09
C-X-C motif chemokine ligand 2 (CXCL2)	4.68	4.08	−0.60	7.37	7.30	−0.07
C-X-C motif chemokine ligand 5 (CXCL5)	3.08	2.72	−0.36	5.75	5.63	−0.11
C-X-C motif chemokine ligand 8 (CXCL8)	3.53	3.03	−0.49	7.87	7.73	−0.13
insulin induced gene 1 (INSIG1)	2.37	1.67	−0.70	4.58	4.47	−0.11
lysyl oxidase (LOX)	1.53	0.27	−1.26	3.58	2.97	−0.61
nephroblastoma overexpressed (NOV)	2.23	0.25	−1.98	2.03	0.83	−1.20
prolyl 4-hydroxylase subunit alpha 2 (P4HA2)	2.29	1.88	−0.40	2.40	1.85	−0.55
Pim-2 proto-oncogene, serine/threonine kinase (PIM2)	3.18	3.00	−0.18	3.78	3.70	−0.07
pentraxin 3 (PTX3)	2.19	1.57	−0.61	5.24	4.50	−0.74
serum/glucocorticoid regulated kinase 1 (SGK1)	1.64	0.38	−1.26	2.18	1.91	−0.27
solute carrier organic anion transporter family member 2A1 (SLCO2A1)	1.94	0.13	−1.81	2.95	2.38	−0.57
solute carrier organic anion transporter family member 4A1 (SLCO4A1)	2.08	1.51	−0.57	5.89	5.23	−0.67
superoxide dismutase 2 (SOD2)	2.62	2.46	−0.15	5.80	5.73	−0.07
tissue factor pathway inhibitor 2 (TFPI2)	2.32	1.12	−1.20	3.80	3.23	−0.58

Inflammatory response genes were selected based on an IL-1β (10 ng/mL)-induced gene expression fold change >1.5 compared to control. Only genes with a uniform expression pattern in both HK-2 and RPTEC/TERT1 cells, that is, inhibition of IL-1β-induced gene expression by empagliflozin (500 nM) in two independent experiments per cell line (*n* = 2, each) are presented.

**Table 2:** Effect of empagliflozin (500 nM) on *SGLT2* gene expression in IL-1β (10 ng/mL)-stimulated HK-2 (A) and RPTEC/TERT1 (B) cells.

A	HK-2 (Experiment 1)			HK-2 (Experiment 2)		
Gene Name	Fold Change to Control		Fold Change to IL-1β	Fold Change to Control		Fold Change to IL-1β
	IL-1β	IL-1β + EMPA	IL-1β + EMPA	IL-1β	IL-1β + EMPA	IL-1β + EMPA
Sodium glucose cotransporter, member 2 (SGLT2) (SLC5A2)	−0.05	−0.1	−0.05	−0.56	−0.59	−0.03
**B**	**RPTEC/TERT1 (Experiment 1)**			**RPTEC/TERT1 (Experiment 2)**		
**Gene Name**	**Fold Change to Control**		**Fold Change to IL-1β**	**Fold Change to Control**		**Fold Change to IL-1β**
	**IL-1β**	**IL-1β + EMPA**	**IL-1β + EMPA**	**IL-1β**	**IL-1β + EMPA**	**IL-1β + EMPA**
Sodium glucose cotransporter, member 2 (SGLT2) (SLC5A2)	−0.31	−0.46	−0.15	−0.26	−0.39	−0.13

**Table 3:** Clustering of annotated pathways (enrichment score 3.64).

Knowledgebase	Annotated Pathway/Term	EASE Score (*p* Value)
INTERPRO	CXC chemokine	2.2 × 10^−7^
INTERPRO	CXC chemokine, conserved site	2.2 × 10^−7^
INTERPRO	chemokine interleukin-8-like domain	1.1 × 10^−5^
SMART	SCY	1.6 × 10^−5^
GOTERM_MF_DIRECT	chemokine activity	1.8 × 10^−5^
UP_KEYWORDS	cytokine	1.9 × 10^−5^
UP_KEYWORDS	disulfide bond	2.8 × 10^−5^
GOTERM_MF_DIRECT	CXCR chemokine receptor binding	3.8 × 10^−5^
GOTERM_BP_DIRECT	cell chemotaxis	4.3 × 10^−5^
GOTERM_BP_DIRECT	chemokine-mediated signaling pathway	5.6 × 10^−5^
UP_KEYWORDS	secreted	1.2 × 10^−4^
KEGG_PATHWAY	salmonella infection	1.9 × 10^−4^
UP_SEQ_FEATURE	signal peptide	2.0 × 10^−4^
GOTERM_BP_DIRECT	positive regulation of neutrophil chemotaxis	2.5 × 10^−4^
KEGG_PATHWAY	cytokine-cytokine receptor interaction	2.7 × 10^−4^
GOTERM_BP_DIRECT	chemotaxis	2.8 × 10^−4^
GOTERM_CC_DIRECT	extracellular region	5.5 × 10^−4^
GOTERM_BP_DIRECT	inflammatory response	6.1 × 10^−4^
GOTERM_BP_DIRECT	response to lipopolysaccharide	6.7 × 10^−4^
GOTERM_BP_DIRECT	immune response	9.0 × 10^−4^
UP_KEYWORDS	signal	9.9 × 10^−4^
GOTERM_CC_DIRECT	extracellular space	1.4 × 10^−3^
KEGG_PATHWAY	chemokine signaling pathway	2.0 × 10^−3^
KEGG_PATHWAY	legionellosis	2.6 × 10^−3^
KEGG_PATHWAY	NOD-like receptor signaling pathway	2.8 × 10^−3^
UP_KEYWORDS	chemotaxis	3.2 × 10^−3^
KEGG_PATHWAY	rheumatoid arthritis	6.8 × 10^−3^
UP_KEYWORDS	inflammatory response	7.7 × 10^−3^
KEGG_PATHWAY	TNF signaling pathway	9.9 × 10^−3^
UP_SEQ_FEATURE	disulfide bond	9.9 × 10^−3^

## Data Availability

The complete microarray data set has been submitted to the Gene Expression Omnibus (GEO) (http://www.ncbi.nlm.nih.gov/geo/) (accession number: GSE155867 and GSE169683).
